# Gene Expression Profiles of Chicken Embryo Fibroblasts in Response to *Salmonella* Enteritidis Infection

**DOI:** 10.1371/journal.pone.0127708

**Published:** 2015-06-05

**Authors:** Ama Szmolka, Zoltán Wiener, Marta Elsheimer Matulova, Karolina Varmuzova, Ivan Rychlik

**Affiliations:** 1 Institute for Veterinary Medical Research, Centre for Agricultural Research, Hungarian Academy of Sciences, Budapest, Hungary; 2 Department of Genetics, Cell and Immunobiology, Semmelweis University, Budapest, Hungary; 3 Veterinary Research Institute, Brno, Czech Republic; Indian Institute of Science, INDIA

## Abstract

The response of chicken to non-typhoidal *Salmonella* infection is becoming well characterised but the role of particular cell types in this response is still far from being understood. Therefore, in this study we characterised the response of chicken embryo fibroblasts (CEFs) to infection with two different *S*. Enteritidis strains by microarray analysis. The expression of chicken genes identified as significantly up- or down-regulated (≥3-fold) by microarray analysis was verified by real-time PCR followed by functional classification of the genes and prediction of interactions between the proteins using Gene Ontology and STRING Database. Finally the expression of the newly identified genes was tested in HD11 macrophages and *in vivo* in chickens. Altogether 19 genes were induced in CEFs after *S*. Enteritidis infection. Twelve of them were also induced in HD11 macrophages and thirteen in the caecum of orally infected chickens. The majority of these genes were assigned different functions in the immune response, however five of them (LOC101750351, K123, BU460569, MOBKL2C and G0S2) have not been associated with the response of chicken to *Salmonella* infection so far. K123 and G0S2 were the only ’non-immune’ genes inducible by *S*. Enteritidis in fibroblasts, HD11 macrophages and in the caecum after oral infection. The function of K123 is unknown but G0S2 is involved in lipid metabolism and in β-oxidation of fatty acids in mitochondria.

## Introduction

Non-typhoid *Salmonella enterica* serovars such as *Salmonella* Enteritidis (*S*. Enteritidis) are some of the most important pathogens causing gastroenteritis in humans. Reservoirs of *S*. Enteritidis for humans can be found in poultry production and in egg-laying hens in particular [1]. Infection of chickens and hens with *Salmonella* serovars other than *S*. Gallinarum usually does not result in any gross clinical signs. However, extensive interactions can be observed in the caecum, which is the most preferred colonisation site of non-typhoid *Salmonella* serovars.

*S*. Enteritidis or *S*. Typhimurium infection of chickens results in moderate inflammation in the caecum accompanied by the induction of inflammatory cytokines such as IL1β, IL8, IL6, IL17 or IL22, followed by a change in the gene expression of leukocytes residing in the intestinal tract and by an infiltration of leukocytes from the circulatory system [2–5]. Inflammation usually disappears 2–3 weeks after the infection of newly hatched chickens [6, 7]. Events occurring at tissue level have been characterised in considerable detail using different genome-wide approaches [6–8]. However, such approaches did not provide information on the contribution of different cell types constituting the whole tissue. Sorted leukocytes, either from the spleen or from the peripheral blood, were therefore used in several studies, which showed that macrophages and heterophils represent key cells that limit *Salmonella* infection to the chicken intestinal tract [2, 8, 9]. However, even during *Salmonella*-induced inflammation, these cells represent minority subpopulations in the caecal tissue. The response of the majority of cell subpopulations in caecal tissue, *e*.*g*. intestinal epithelial cells or fibroblasts, is less clear. At least the epithelial cells were shown to respond to *Salmonella* infection and contribute to the overall immune response, and play a central role in response to *Salmonella* Enteritidis during the acute phase and carrier-state in chicken [10, 11]. In this study we therefore describe the interaction of fibroblasts with *S*. Enteritidis.

Fibroblasts are ubiquitous non-phagocytic cells with a long lifespan and play an important role in inflammation and tissue repair. Chicken fibroblasts are widely used for the studying the interactions between chickens and avian viruses [12, 13], but their role in the response to *Salmonella* has been poorly characterised [14]. Garcia Del Portillo et al. [15] regarded fibroblasts as potential host cells during *Salmonella* infection. The same group of researchers also described unusual properties of an otherwise attenuated *phoP* mutant of *S*. Typhimurium exhibiting an intracellular overgrowth phenotype in rat fibroblasts [16]. Chicken fibroblasts can be invaded by both pathogenic *E*. *coli* and *S*. Typhimurium and respond to stimulation with heat-killed *S*. Typhimurium by the induction of TLR15 expression [14]. All of these findings have indicated that fibroblasts participate in the interaction between *Salmonella* and the host. However, their response to *Salmonella* infection has never been characterised in genome-wide studies.

This is why we were interested whether fibroblasts respond to *S*. Enteritidis infection and to what extent their response differs from that of other cells and caecal tissue. To address this, we characterised the gene expression of chicken embryo fibroblasts (CEFs) after infection with two different *S*. Enteritidis strains using microarray analysis. Results from the microarray analysis were verified with quantitative real-time PCR in fibroblasts, in the HD11 macrophage-like cell line and *in vivo* in chickens after infections. Besides the re-identification of genes coding for multiple cytokines and chemokines, G0S2 protein was found to be inducible by *S*. Enteritidis infection. G0S2 protein is involved in the control of lipid availability for β-oxidation of fatty acids in mitochondria, which may affect production of reactive oxygen species during inflammatory response.

## Materials and Methods

### Ethics statement

Chicken embryo fibroblast (CEF) cell cultures were purchased from the Virology Laboratory, Veterinary Diagnostic Directorate of the National Food Chain Safety Office of Hungary, where freshly prepared cultures of CEFs are routinely used for virus isolation. The handling of chicken embryos was performed in accordance with the relevant Hungarian legislation (Animal Protection and Welfare Act No. 103/2002). The handling of animals in the study was performed in accordance with the current Czech legislation (Animal Protection and Welfare Act No. 246/1992 Coll. of the Government of the Czech Republic). The specific experiments were approved by the Ethics Committee of the Veterinary Research Institute (permit number 48/2010), followed by approval by the Committee for Animal Welfare of the Ministry of Agriculture of the Czech Republic (permit number MZe 1226). No mortality was registered during the whole period of the chicken infection experiments.

### Bacterial strains

Wild-type strains of *Salmonella* Enteritidis 147 [17] and *S*. Enteritidis 11 [18] of poultry origin were used in this study. Bacteria were grown in Tryptic Soy Broth (TSB, Sigma-Aldrich) for 16 h at 37°C prior the infection of CEFs.

### *Salmonella* infection of CEFs

CEFs were freshly prepared from 12-day-old chicken embryos of the Leghorn breed and maintained in MEM (Sigma-Aldrich) with 5% fetal calf serum (FCS) for ~ 24 hours. The day before infection, CEFs were seeded into 36 mm Petri dishes (Nunc) and grown for 18 hours at 37°C under 5% CO_2_. The purity of the cell population was tested by stereo microscopy, showing that fibroblast cells formed ~90% of the isolated cells. On the second day of growth, semi-confluent cell cultures were washed three times with HBSS (Sigma-Aldrich) and MEM was replaced with DMEM (Sigma-Aldrich) with 5% fetal calf serum and 1% D-mannose. CEFs were infected for 4 h at 37°C and 5% CO_2_ with overnight bacterial cultures at a multiplicity of infection (MOI) equal to 10. After the incubation, CEFs were washed 3× with HBSS and lysed directly in a cell-culture vessel by adding 600 μl RLT buffer from the RNA purification kit (see below). Infection with each of the strains was performed in four replicates.

### Microarray workflow and data analysis

The total RNA was extracted from the fibroblasts using a RNeasy Mini Kit according to the manufacturer’s instructions (Qiagen). One μg of total RNA was transcribed to cDNA with a Low-input RNA Linear Amplification Kit (Agilent Technologies) and then transcribed to cyanine-3 (Cy3)-labelled cRNA according to the One-Color Microarray-Based Gene Expression Manual v5.5 (Agilent Technologies). The fluorescent cRNA probes were purified using the RNeasy Mini Kit (Qiagen), and dye incorporation was determined with a NanoDrop ND-1000 (Thermo Scientific).

Six hundred ng of Cy3-labeled cRNA were hybridised to Agilent chicken custom 8×15K microarrays. In total, 13,681 probes were designed to characterise the expression of ~9,000 transcripts of *Gallus gallus* (S1 Table). Hybridisation was performed overnight at 65°C. The slides were washed, treated with Stabilizing and Drying Solution (Agilent Technologies) and scanned with an Agilent DNA Microarray Scanner (Agilent Technologies). Feature Extraction software 9.1 was used for image analysis. Data analysis was performed using BRB-Array Tools (Biometric Research Branch). Fluorescent signal was normalised to GAPDH and 28S rRNA. Only genes with a fold change ≥3 and a P value < 0.05 were considered for further analyses.

Microarray datasets about the CEF infection experiment have been deposited in NCBI’s Gene Expression Omnibus (GEO) database. The corresponding accession numbers are: platform: GPL19971; series: GSE67459.

Functional classification was performed using the STRING Database v9.1 [19]. This database was used both for Gene Ontology (GO) classification and to search for potential interactions among newly identified genes. For functional classification only significant GO enrichments at P < 0.05 were considered.

### Quantitative reverse transcriptase PCR

Expression of genes with a fold change of ≥3 identified by microarray analysis was verified using quantitative real-time PCR. Ten ng of total fibroblast RNA was reverse-transcribed into cDNA using an iScript cDNA Synthesis Kit (Bio-Rad) and oligo (dT) primers. Primers for real-time PCR were designed using the Primer3 software and are listed in S2 Table. The real-time PCR reaction was performed in 3 μl volumes in 384-well microplates using a QuantiTect SYBR Green RT-PCR Master Mix (Qiagen) and a Nanodrop II Stage pipetting station (Innovadyne) for PCR mix dispensing. Amplification and signal detection were performed using a LightCycler II (Roche) with an initial denaturation at 95°C for 15 min followed by 40 cycles of 95°C for 20 s, 60°C for 30 s and 72°C for 30 s. Each sample was subjected to real-time PCR in triplicate. The Ct values of the genes of interest were normalised (ΔCt) to an average Ct value of three housekeeping genes (glyceraldehyde-3-phosphate dehydrogenase, TATA-binding protein and ubiquitin) and the relative expression of each gene of interest was calculated as 2^-ΔCt^. These expression levels were used for statistical analyses by Kruskal-Wallis non-parametric test with Dunn’s multiple comparison test. Finally, fold inductions between the experimental and control groups were calculated.

### Infection of HD11 macrophages

Chicken macrophage-like cell line HD11 was cultured at 37°C under 5% CO_2_ in RPMI-1640 (Sigma-Aldrich). *S*. Enteritidis 147 was grown statically in LB broth at 37°C for 18 hours. This culture was diluted 800× in LB broth and incubated for an additional 4 hours with aeration at 37°C to obtain bacteria in the late logarithmic growth phase of a highly invasive phenotype. Prior to infection of HD11, the bacteria were pelleted by centrifugation (10 min at 6,500 ×g) and resuspended in PBS to OD = 0.3. HD11 cells were infected with *S*. Enteritidis at a multiplicity of infection equal to 1 for 1 h. Free bacteria were washed away and gentamicin was added to fresh medium (100μg/ml) to kill extracellular bacteria. One hour later, the medium was replaced with fresh medium containing 15 μg/ml gentamicin to prevent multiplication of extracellular bacteria that were eventually released during cultivation from dead cells. Two hours later, *i*.*e*. 4 hours after the infection of HD11 cells, the wells were treated with TRI Reagent (Molecular Research Center) for RNA purification. The whole experiment was performed on two independent occasions in triplicates in each of the experiments.

### *In vivo* expression

In the first experiment, 4 newly hatched chickens per group were orally inoculated with 0.1 mL of wild-type *S*. Enteritidis 147. The infectious dose was approx. 10^7^ CFU and the infected chickens were euthanised 4 days post infection. The control group consisted of 4 non-infected chickens euthanised on day 5 of life. In the second experiment, four 42-day-old chickens were intravenously infected with 10^7^ CFU of *S*. Enteritidis in 0.1 ml of PBS and euthanised 4 days post infection. Four 46-day-old non-infected chickens were included as negative controls. During necropsies, approx. 30 mg of the caecum or spleen were collected from each chicken, placed into RNALater (Qiagen) and stored at -70°C. Prior to purification, the tissue was homogenised using MagnaLyzer (Roche) and RNA was purified with the RNeasy Mini Kit (Qiagen). One μg of total RNA was immediately transcribed with M-MLV reverse transcriptase (Invitrogen) and oligo (dT) primers into cDNA, and real-time PCR was performed as described above.

## Results

### Response of CEFs to *S*. Enteritidis infection

**Fig 1:**
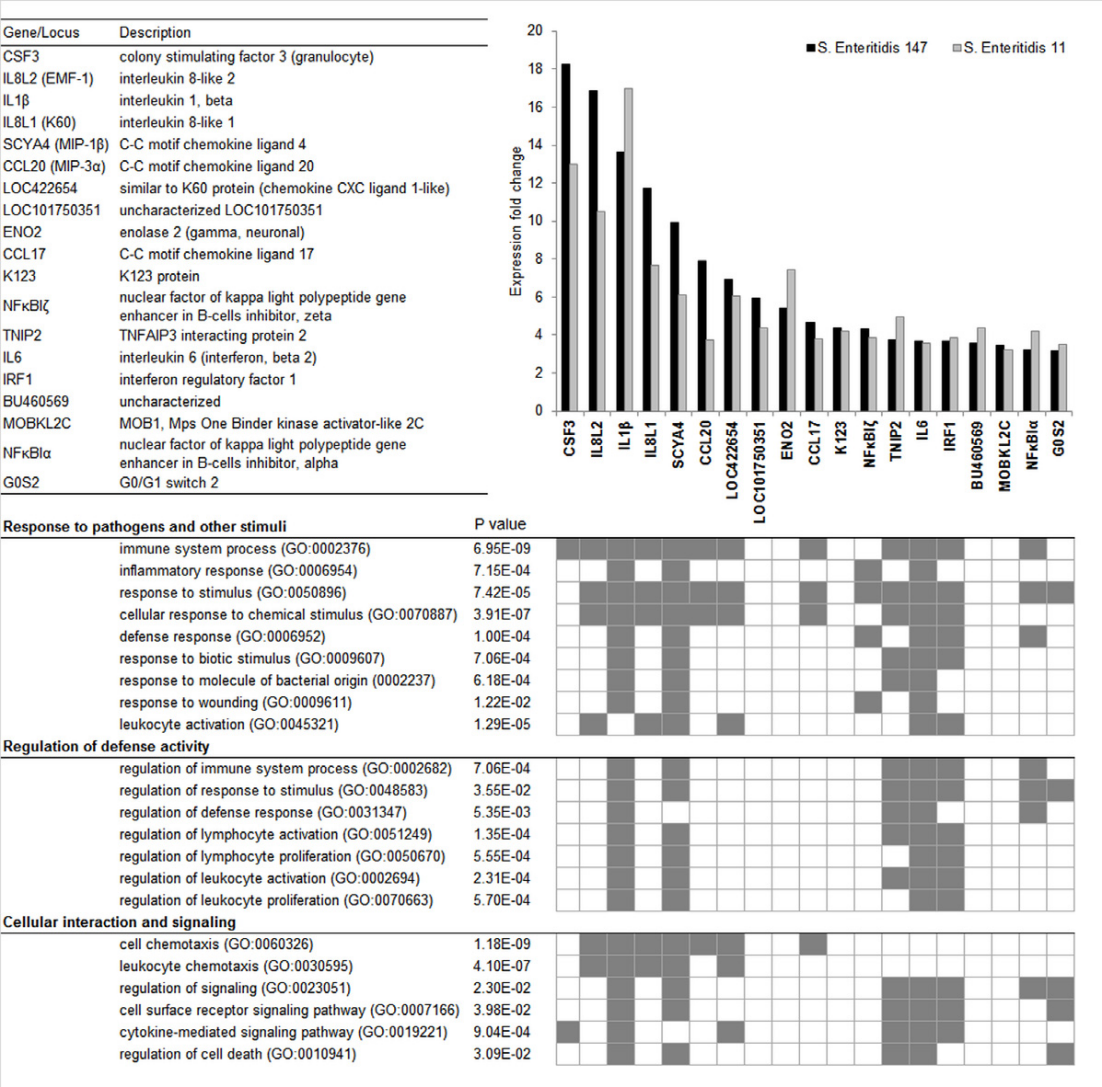
Functional classification of genes induced in CEFs after infection with wild-type *S*. Enteritidis. Genes are ranked in descending order of their expression fold change. Functional annotation of genes was performed with the STRING database v9.1. and is represented by gene ontology (GO) terms for biological process (BP).

CEFs responded to infection with at least one of the *S*. Enteritidis strains by a significant change in the expression of 127 genes (S3 Table). However, only 41 genes were differentially expressed in both experimental groups. Out of these, 22 genes were significantly up-regulated and 19 were significantly down-regulated. The expression of these genes was verified with real-time PCR. The data obtained by real-time PCR did not confirm the suppression of any of the dow-nregulated genes identified by microarray analysis but confirmed a significant induction of 19 up-regulated genes (Fig 1).

### Functional classification of significantly inducible genes in CEFs

**Fig 2:**
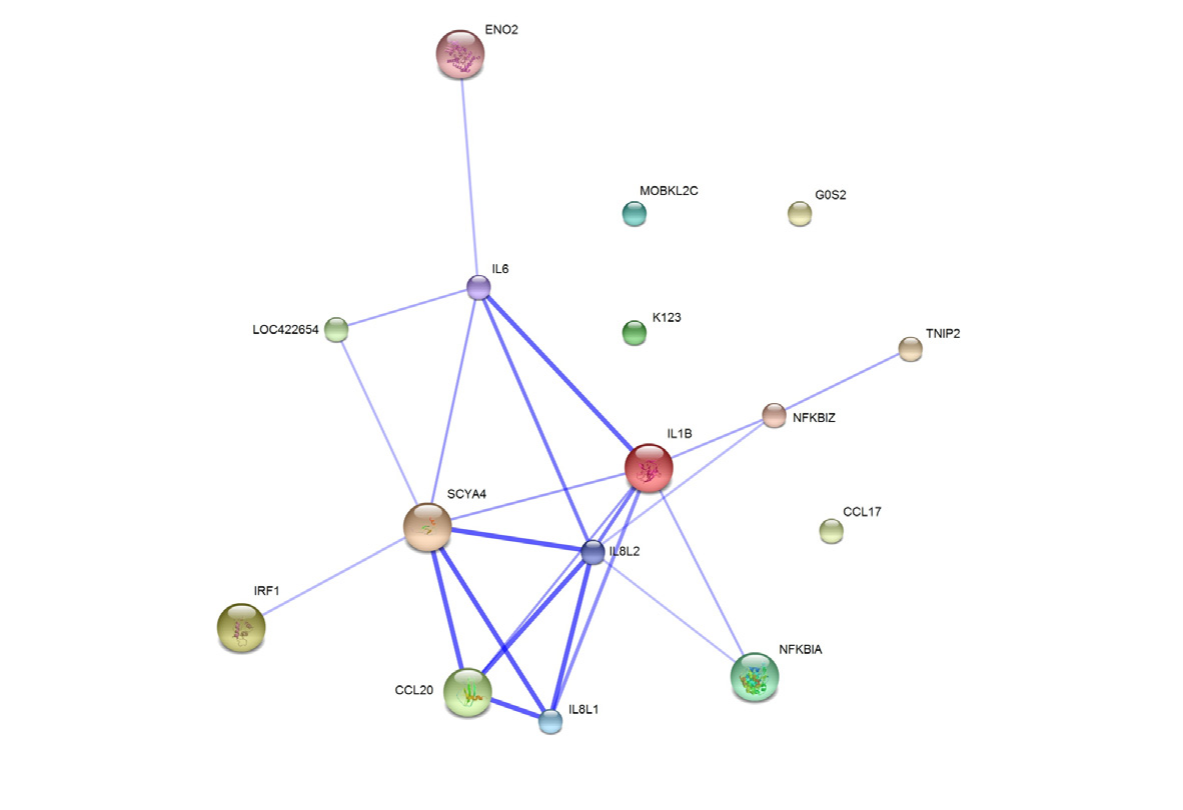
Interaction analysis of genes inducible in CEFs infected with *S*. Enteritidis. Figure presents a confidence view of protein interactions in chicken (*Gallus gallus*) generated by the STRING Database v9.1 for genes significantly upregulated more than threefold in CEFs in response to both *Salmonella* strains. Lines represent associations based on experimental data, co-expression, databases and/or homology.

Two different approaches, *i*.*e*. Gene Ontology (GO) classification and STRING database search, were used for the functional classification of the 19 genes identified as inducible in CEFs by both microarray analysis and real-time PCR. Based on GO classification of biological processes, these genes were grouped into three main functional clusters: Response to pathogens and other stimuli, Regulation of defense activity, and Cellular interaction and signalling. Only 6 genes (LOC101750351, ENO2, K123, BU460569, MOBKL2C and G0S2) were not assigned any function associated with immune response, except for G0S2 function in “response to stimulus” (Fig 1). STRING database search showed that the interactions among the majority of these genes have already been reported. However LOC101750351, CCL17, K123, BU460569, MOBKL2C and G0S2 have never been described in association with any of the remaining genes identified in this study (Fig 2).

### Expression of selected genes in chicken HD11 macrophages

In the next experiment we tested whether the genes identified as inducible in CEFs were also inducible in HD11 macrophages. CSF3, IL8L2 (EMF-1), IL1β, IL8L1 (K60), SCYA4, CCL20, CCL17, K123, NFκBIζ, IL6, NFκBIα and G0S2 were significantly induced also in HD11 macrophages. The remaining 7 genes, *i*.*e*. LOC422654, LOC101750351, ENO2, TNIP2, IRF1, BU460569 and MOBKL2C were not significantly induced in HD11 macrophages (Table 1). Six of them could therefore be considered as specific for CEFs in comparison to macrophages, as IRF1 was shown to be induced also in RAW264.7 murine macrophage cells in contact to *Salmonella* [20].

**Table 1:** Genes induced in CEFs, HD11 macrophages and chicken tissues after *S*. Enteritidis infection.

Gene/Locus	Description	Chicken embryo fibroblast				HD11 macrophage	Tissue	
		Microarray		qPCR		qPCR	qPCR	
		SE 147	SE 11	SE 147	SE 11	SE 147	SE 147	SE 147
		4 h	4 h	4 h	4 h	4 h	Caecum	Spleen
CSF3	colony stimulating factor 3 (granulocyte)	18.26 *	**12.99**	**18.47**	**13.53**	**16.70**	**81.85**	**24.22**
IL8L2 (EMF-1)	interleukin 8-like 2	**16.84**	**10.49**	**16.36**	**12.67**	**16.71**	**3.45**	0.71
IL1β	interleukin 1, beta	**13.62**	**16.96**	**15.19**	**22.08**	**11.56**	**41.03**	**7.98**
IL8L1 (K60)	interleukin 8-like 1	**11.70**	**7.65**	**14.10**	**9.16**	**6.92**	2.19	0.63
SCYA4 (MIP-1β)	C-C chemokine ligand 4	**9.95**	**6.09**	**13.67**	**7.94**	**4.91**	**23.68**	**7.37**
CCL20 (MIP-3α)	C-C motif chemokine ligand 20	**7.89**	**3.75**	**6.86**	**3.46**	**16.87**	**7.39**	**11.86**
CCL17	C-C motif chemokine ligand 17	**4.65**	**3.77**	**2.49**	**2.34**	**2.27**	0.65	0.31
K123	K123 protein	**4.36**	**4.20**	**5.50**	**4.69**	**6.43**	**10.68**	1.22
NFκBIζ	nuclear factor of kappa light polypeptide gene enhancer in B-cells inhibitor, zeta	**4.34**	**3.84**	**5.97**	**4.15**	**3.50**	1.24	1.21
IL6	interleukin 6 (interferon, beta 2)	**3.70**	**3.57**	**6.19**	**6.17**	**454.54**	**137.01**	**18.75**
NFκBIα	nuclear factor of kappa light polypeptide gene enhancer in B-cells inhibitor, alpha	**3.21**	**4.19**	**4.00**	**5.71**	**2.85**	**3.07**	1.07
G0S2	G0/G1 switch 2	**3.13**	**3.52**	**17.47**	**24.83**	**2.41**	**9.18**	0.93
LOC422654	similar to K60 protein (chemokine CXC ligand 1-like)	**6.91**	**6.08**	**8.38**	**8.24**	0.79	3.11	0.24
LOC101750351	uncharacterized	**5.95**	**4.39**	**6.91**	**4.90**	1.17	0.91	1.27
ENO2	enolase 2 (gamma, neuronal)	**5.42**	**7.44**	**2.91**	**2.40**	0.41	**3.64**	**4.72**
TNIP2	TNFAIP3 interacting protein 2	**3.73**	**4.96**	**3.98**	**4.52**	1.38	**3.53**	1.20
IRF1	interferon regulatory factor 1	**3.67**	**3.84**	**4.21**	**4.84**	1.89	**4.91**	1.74
BU460569	uncharacterized	**3.59**	**4.36**	**3.34**	**2.87**	1.13	1.46	0.85
MOBKL2C	MOB1, Mps One Binder kinase activator-like 2C	**3.42**	**3.21**	**3.54**	**3.20**	1.65	**3.29**	1.38

Values in the table represent fold inductions to appropriate non-infected control.

* Bold numbers represent fold inductions higher than twofold and significantly different from non-infected controls at p < 0.05.

### *In vivo* expression of selected genes

In the last two experiments we tested whether the genes inducible in CEFs were also inducible *in vivo* in chickens following oral and intravenous infection with *S*. Enteritidis. Thirteen genes inducible in CEFs were also induced in the caecum after oral infection and the induction was not confirmed in only 6 genes, *i*.*e*. IL8L1 (K60), CCL17, NFκBIζ, LOC422654, LOC101750351 and BU460569. Intravenous infection led to a significant induction of 6 out of 19 genes in the spleen (Table 1).

## Discussion

As a response of chicken embryo fibroblasts to 4 hour stimuli with *S*. Enteritidis 19 genes were identified as inducible at a fold change ≥3. None of the genes inducible in CEFs coded for effector proteins involved in pathogen inactivation such as lysozyme, ExFABP or other antimicrobial peptides, *e*.*g*. cathelicidins or gallinacins [7]. Instead, genes such as IL1β, IL6 or both chicken orthologues of IL8 (EMF-1 and K60), which are commonly used as markers of inflammation following *Salmonella* infection of chickens [3–5] were inducible also in fibroblasts. This made the response of fibroblasts similar to that of epithelial cells in terms of the presence of the corresponding genes [11]. Induction of transcription factors NFκBIα and NFκBIζ was also consistent with fibroblast inflammatory signalling [21–23]. The absence of comparative data on CEFs infected with *Salmonella* and the reduced number of inducible genes may lead to the conclusion that fibroblasts are not the most important cells in the host interaction with *S*. Enteritidis at the selected time point of gene expression analysis. However, due to their numerical dominance in the caecal mucosa, infected fibroblasts can considerably affect cytokine signalling and total gene expression in the chicken caecum. This conclusion is further supported by the fact that 13 out of 19 genes significantly induced in CEFs were induced also in the chicken caecum following *S*. Enteritidis infection.

Six of the genes identified in this study have not been assigned any function in the immune response so far. Out of these, LOC101750351, BU460569, MOBKL2C and ENO2 could be specific for CEFs as they were not induced in HD11 cells. The functions of LOC101750351 and BU460569 are completely unknown. MOBKL2C codes for a kinase activator, for which the human homologue is acting as a tumour suppressor [24]. ENO2 codes for enolase converting 2-phosphoglycerate to phosphoenolpyruvate. Its induction may indicate a higher rate of glycolysis inside infected cells. LOC101750351, BU460569, MOBKL2C and ENO2 may therefore potentiate basic cell functions of CEFs, and since these genes are not inducible in HD11 macrophages, they may not contribute to the immune response.

K123 and G0S2 were the only ‘non-immune’ genes equally inducible in CEFs, HD11 macrophages and in the caecum after oral infection indicating that, although annotated as being rather related to several other functions like stress, metabolism than to the immune response, they may have functions in immunity. Interestingly, these genes were not induced in the spleen following intravenous infection. The function of K123 is unknown, however the InterPro database predicted that it may have a non-specific DNA/RNA endonuclease activity. G0S2 is involved in lipid metabolism and in β-oxidation of fatty acids in mitochondria [25]. *Gallus gallus* is defective in myeloperoxidase and in alternative pathways suggesting that pathogen inactivation must therefore be active in chicken phagocytes. This includes the induction of IRG1, itaconic acid synthase with an indirect effect on the production of reactive oxygen species as respiration by-products [26–28]. Another example is ExFABP, an extracellular fatty acid binding protein inducible following *S*. Enteritidis infection [29, 30], which may provide fatty acids for mitochondrial respiration during infection. Interestingly, neutrophil phagosomes in humans were found to contain elevated levels of mitochondrial proteins, which may indicate fusions of phagosomes with mitochondria resulting in the release of mitochondrial reactive oxygen species into maturing phagosomes [31]. The induction of G0S2, however, should decrease the availability of lipids and fatty acids for mitochondrial oxidation and thus decrease mitochondrial activity and reactive oxygen species production [25]. In the case of fibroblasts, this may lead to apoptosis and the release of intracellular *Salmonella*. In the case of macrophages, this might be necessary for preserving a balance between extensive respiration producing reactive oxygen species and damage to cells. This hypothesis is in agreement with our preliminary observations indicating that maximal G0S2 expression in the chicken caecum following *S*. Enteritidis infection is observed 8–12 days after the infection of newly hatched chickens (unpublished data). G0S2 may therefore represent a protein which allows the release of intracellular *Salmonella* from non-professional phagocytes during recovery from infection and control of respiratory burst by phagocytes, thus decreasing unnecessary damage to the host’s tissues.

## Supporting Information

S1 Table Selected data from the microarray analysis of CEFs, with a focus on transcripts representing *Gallus gallus* only. Up- and down-regulated transcripts are listed in descending order of their expression fold change.
(XLSX)

S2 Table List of RT PCR primers used in this study. (XLSX)

S3 Table Basic data from all *in vitro* and *in vivo* experiments. A grey background indicates significant misregulation which passed through threshold criteria, *i*.*e*. more than threefold misregulation in the microarray analysis or twofold misregulation in RT PCR.
(XLSX)
